# Motor-Cognitive Neural Network Communication Underlies Walking Speed in Community-Dwelling Older Adults

**DOI:** 10.3389/fnagi.2019.00159

**Published:** 2019-07-16

**Authors:** Victoria N. Poole, On-Yee Lo, Thomas Wooten, Ikechukwu Iloputaife, Lewis A. Lipsitz, Michael Esterman

**Affiliations:** ^1^Center for Translational Research in Mobility & Falls, Hinda and Arthur Marcus Institute for Aging Research, Hebrew SeniorLife, Boston, MA, United States; ^2^Beth Israel Deaconess Medical Center, Boston, MA, United States; ^3^Harvard Medical School, Boston, MA, United States; ^4^Neuroimaging Research for Veterans (NeRVe) Center, VA Boston Healthcare System, Boston, MA, United States; ^5^Geriatric Research, Education, and Clinical Center (GRECC), VA Boston Healthcare System, Boston, MA, United States; ^6^Department of Psychiatry, Boston University, Boston, MA, United States

**Keywords:** older adults, gait speed, dual-task cost, resting-state fMRI, functional connectivity

## Abstract

While walking was once thought to be a highly automated process, it requires higher-level cognition with older age. Like other cognitive tasks, it also becomes further challenged with increased cognitive load (e.g., the addition of an unrelated dual task) and often results in poorer performance (e.g., slower speed). It is not well known, however, how intrinsic neural network communication relates to walking speed, nor to this “cost” to gait performance; i.e., “dual-task cost (DTC).” The current study investigates the relationship between network connectivity, using resting-state functional MRI (rs-fMRI), and individual differences in older adult walking speed. Fifty participants (35 females; 84 ± 4.5 years) from the MOBILIZE Boston Study cohort underwent an MRI protocol and completed a gait assessment during two conditions: walking quietly at a preferred pace and while concurrently performing a serial subtraction task. Within and between neural network connectivity measures were calculated from rs-fMRI and were correlated with walking speeds and the DTC (i.e., the percent change in speed between conditions). Among the rs-fMRI correlates, faster walking was associated with increased connectivity between motor and cognitive networks and decreased connectivity between limbic and cognitive networks. Smaller DTC was associated with increased connectivity within the motor network and increased connectivity between the ventral attention and executive networks. These findings support the importance of both motor network integrity as well as inter-network connectivity amongst higher-level cognitive networks in older adults’ ability to maintain mobility, particularly under dual-task (DT) conditions.

## Introduction

Walking speed is now widely accepted as a clinically meaningful marker of general function and wellbeing in older adults (Cesari et al., 2005; Afilalo et al., 2010; Verghese et al., 2011). For this reason, it is frequently measured within the geriatric clinic and has been referred to as the “sixth vital sign” (Fritz and Lusardi, 2009; Middleton et al., 2015). However, many factors contribute to declines in walking speed as we age (Tiedemann et al., 2005). For instance, although walking slows with musculoskeletal and peripheral nervous system disorders, it also slows with central nervous system disorders (Hajjar et al., 2009). Previous studies support the premise that walking engages several neurocognitive and neurovascular processes, including gait planning and initiation, sensory-motor integration, memory, the ability to detect and accommodate altered peripheral neuromusculoskeletal function and neurovascular coupling (Halliday et al., 1998; Jahn et al., 2004, 2008; Seidler et al., 2010; Sorond et al., 2011; Takakusaki, 2017).

The *cognitive* nature of walking is particularly important for “real-world” mobility. With or without physical limitations, successful mobility in living environments requires higher-level cognitive processes like attention (Woollacott and Shumway-Cook, 2002), executive functioning (Yogev-Seligmann et al., 2008), self-reference (Sholl, 2001), motor control (Winter, 2009), and caution (Donoghue et al., 2013). A person must constantly process the information within his or her environment to navigate within it, often whilst performing additional tasks, like talking or texting. When attempting to perform these additional tasks, the brain must shift and prioritize attention (Yogev-Seligmann et al., 2008). This typically results in a “cost” of slower speed (or poorer task performance) and has been associated with an increased risk of falls (Beauchet et al., 2008), accidents (Neider et al., 2011), and other adverse outcomes (Montero-Odasso et al., 2017). To study this real-world phenomenon, both normal walking and dual-task (DT) paradigms have been implemented within the research laboratory (Verhaeghen et al., 2003) and several studies have uncovered relationships between abnormal walking characteristics and poorer cognition (Li et al., 2018), as measured by cognitive and neuropsychological assessments.

Although these findings provide a clear basis for the premise that higher levels of neurocognitive function underlie normal gait, limitations exist in the actual study of brain function. Most studies have either utilized wearable techniques, like functional near-infrared spectroscopy (fNIRS), or have employed gait imagery paradigms (Hamacher et al., 2015) to study gait, since it is impossible to directly assess walking as a task within the MRI scanner. In addition to brain areas traditionally associated with locomotion (e.g., M1, SMA, premotor cortex, and cerebellum), several studies implicate higher-order and frontoparietal areas in walking (Hanakawa et al., 1999; Allali et al., 2013; Doi et al., 2013; Blumen et al., 2014; Jor’dan et al., 2017). However, far fewer studies have used fMRI to relate stable properties of brain function and communication to individual differences in walking speed. Yuan et al. (2015) were the first to investigate resting-state fMRI (rs-fMRI) associations and found that the connectivity within the sensorimotor, visual, vestibular, and left frontoparietal (i.e., executive control) networks were associated with normal walking and walking while talking. Additionally, Lo et al. (2017) have shown walking relationships within the frontoparietal and attention networks in cognitively impaired older adults.

More studies are needed that explicitly characterize how individual differences in connectivity within and across brain networks relate to “normal” walking, DT walking, and associated dual-task costs (DTCs). To address this need, we have utilized rs-fMRI in community-dwelling older adults from the MOBILIZE Boston Study to investigate normal walking and walking during serial subtraction. We hypothesized that greater within and between sensorimotor network connectivity would be associated with faster preferred walking speed. DT walking (and cost), however, would be *further* associated with the brain network interactions that are related to executive function and attention, as they are more engaged by walking while performing the dual serial subtraction cognitive task.

## Materials and Methods

### Participants

Seventy-six older adults (50 females, 84.5 ± 4.3 years) were recruited from the Maintenance of Balance, Independent Living, Intellect and Zest in the Elderly of Boston (MOBILIZE Boston Study; MBS) cohort to undergo a gait assessment and MRI protocol. Original recruits to the MBS were Boston area residents 70 + years of age (or >65 if living with an already enrolled participant), able to walk 20 feet without personal assistance, had no history of neurological, mental illness, or stroke, and at least a 12th grade education. Mini-Mental State Examination scores collected two years prior to MRI ranged from 19 to 30. A full description of the greater MBS protocol is provided elsewhere (Leveille et al., 2008). To participate in the current study, all participants were further required to perform a gait assessment while simultaneously meeting eligibility criteria for a 60-min MRI. The Hebrew SeniorLife and VA Boston Healthcare System institutional review boards approved this protocol and written consent was required prior to study participation.

### Study Design

Participants took part in two visits. The first visit to the Hebrew SeniorLife Clinical Research Center included a medical history evaluation and gait assessment, where participants were asked to walk over a 16-foot GaitRite (CIR Systems Inc., Havertown, PA, USA) mat during each of two conditions: quietly and during a serial subtraction task. For the preferred walking condition, participants made six separate passes at their preferred pace without interruption, starting and ending approximately 4 feet from the mat. For the DT condition, participants walked while verbally subtracting threes from a randomly given 3-digit number. Walking speed for both conditions was derived from the GaitRite-measured timing and location of individual steps in meters per second (m/s). The primary outcomes were the mean gait speed for each condition calculated by averaging across passes and normalizing by participant height. DTC was calculated as the percent change in speed relative to the quiet condition, such that the higher the cost, the slower the serial subtraction walk. Serial-subtraction task accuracy was not assessed.

Approximately 10 days later, participants completed an MRI protocol at the VA Boston Healthcare System. During this session, participants performed a resting-state scan, during which they were asked to keep their eyes open. A gradient-echo echo-planar sequence was performed with the following parameters: TR = 3,000 ms, TE = 26 ms, flip angle = 90°, 34 slices at 1.5 mm, 64 × 64 matrix, and 120 volumes. A T1-weighted MPRAGE scan (T1 = 1,000 ms, TR = 2.73 ms TE = 3.31 ms, flip angle = 7°, 128 slices at 1.3 mm thickness, 256 × 256 matrix) was also collected for whole-brain high-resolution anatomy. These neuroimaging sessions were performed with two 3T Siemens MRI scanners (TIM Trio, *n* = 18; Prisma^Fit^, *n* = 32) using a 12-channel head coil. To account for potential inter-scanner differences, scanner was included as a covariate in statistical models.

### Functional MRI

Functional data were processed using AFNI (Cox, 1996). Pre-processing steps included: the removal of the first three volumes, time-shifting, volume registration, alignment to high-resolution anatomy, warping into Talairach space, 8-mm kernel smoothing, resampling to 3 × 3 × 3 mm resolution, and scaling to a percentage of the mean. Data were then band-pass filtered from 0.01 to 0.08 Hz and entered into a general linear model to remove the effects of 6° of motion, their derivatives, nuisance CSF, white matter, and global signal. Time points were censored and participants were excluded for excessive motion if they demonstrated greater than 0.5 mm in sudden movement for more than 20% of the scan.

A previously-defined cortical parcellation was then applied to the whole-brain GLM residuals, representing the “cleaned” time series. Briefly, an atlas from Schaefer et al. (2018) was used to parse the cortex into 100 regions that were co-registered with the seven functionally-connected cortical networks identified by Yeo et al. (2011). The seven included the visual (VIS; 17 regions), sensorimotor (SOM; 14 regions), dorsal attention (DAN; 15 regions), ventral attention (VAN; 12 regions), limbic (LIM; 5 regions), executive control (ECN; 13 regions), and default mode (DMN; 24 regions) networks. The average time series were extracted from each brain region and correlated in pairs for a total of 4,950 possible pairwise correlations. To calculate both within and between functional connectivity measures at the network-level, the above estimates were Fisher’s *z*-transformed, grouped, and averaged according to their within- and between-network pairs. This resulted in a total of seven within- and 21 between-network estimates for use in linear regression analyses.

### Statistical Analyses

A matched-pairs *t*-test was conducted to evaluate the change in walking speed between the normal and DT conditions. Non-neural characteristics (e.g., age, sex, body mass index (BMI), type 2 diabetes, hypertension, and arthritis) suspected to influence walking outcomes were evaluated using simple linear regression and rank sums tests. The significant covariates were then included in multiple linear regression models to predict: normal walking speed, DT speed, and the resulting cost from the average network pairs, along with scanner assignment.

Since we performed 28 network-pair analyses for each of the three conditions, we then utilized a permutation procedure to determine the probability of our observed number of significant gait-brain associations. This was done by randomization of individual walking speeds and clinical characteristics, conducting the multiple linear model for each network pair, and determining the number of chance “significant” (i.e., *p* = 0.05) associations. This was repeated for a total of 10,000 iterations for each outcome. All analyses were performed using MATLAB (2014a; Mathworks, Natick, MA, USA) and R (R Core Team, 2018).

## Results

Of the 76 participants that completed the MRI protocol, 50 were included in the analyses. Reasons for exclusion included excessive motion (*n* = 8), missing data or incomplete scan (*n* = 5), reported stroke or incidental findings (*n* = 6), and a score of less than 25 on their most recent MMSE administration (*n* = 7). Participant demographics, clinical characteristics, and gait measurements for the final subset are listed in Table 1.

**Table 1:** Participant demographics and clinical characteristics.

	Study Sample
*N*	50
Female (%)	70
Age (years)	84 ± 5
Mini-Mental State Exam^†^	27 ± 1
Body Mass Index (BMI)	25 ± 5
Hypertension (%)	48
High Cholesterol (%)	52
Diabetes (%)	6
Arthritis (%)	54
*Walking Assessment*^‡^
Velocity_Pref_ (m/s)	1.1 ± 0.3 (0.6, 1.8)
Velocity_Pref norm_	0.017 ± 0.004 (0.007, 0.027)
Velocity_DT_ (m/s)	0.9 ± 0.3 (0.3, 1.5)
Velocity_DT norm_	0.014 ± 0.004 (0.005, 0.023)
DT Cost (%)	19 ± 11 (2.9, 51.8)

Data are expressed in mean ± standard deviation or percentage. ^†^Most recent scores were collected within approximately 2 years of gait and MRI assessment. ^‡^Preferred (Pref) and dual-task (DT) walking outcomes are reported as mean ± SD (range), with and without normalizing by height (inches^−^^1^).

### Gait Assessment

Though participants walked relatively fast at their preferred pace (1.1 ± 0.3 m/s), they walked significantly slower during the serial subtraction task (0.9 ± 0.3 m/s; matched pairs *t*_(49)_ = 13.6, *p* < 0.0001). This resulted in a DTC of 19 ± 11%.

Walking outcomes were then associated with clinical characteristics for covariate consideration. As suspected, participant age was associated with both preferred (*β* = −0.45, *p* = 0.001) and DT walking speeds (*β* = −0.44, *p* < 0.002) and tended to be associated with DTC (*β* = 0.26, *p* < 0.07). No other significant associations with clinical variables (see “Materials and Methods” section) were found.

### Resting-State fMRI

Multiple uni-network linear regression analyses were then performed to assess walking associations with within-network (*n* = 7 networks) and between-network (*n* = 21 network pairs) connectivity, after adjusting for the effects of scanner and participant age. In two outcomes, the number of observed significant gait-brain associations exceeded the probability of chance based on the randomization procedure: 9 of 28 network-pair models were associated with preferred walking (*p* = 0.0027) and 10 were associated with DT walking (*p* = 0.0011) speeds. Only two models were associated with DTC (*p* = 0.36), i.e., 36% of randomized iterations had two or more significant correlations by chance. These models are characterized below.

#### Within-Network Associations

**Figure 1:**
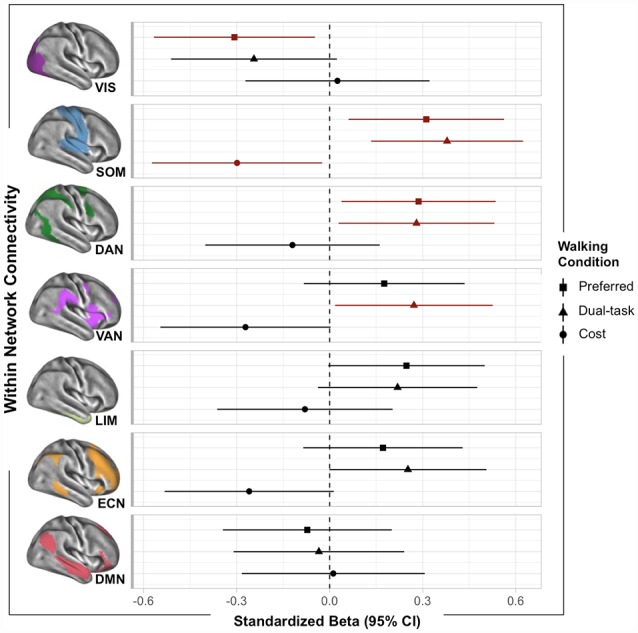
Plot of within-network functional MRI (fMRI) associations with walking outcomes. Standardized betas (95% CI) were extracted from multiple linear regressions adjusted for age and scanner. Points are color-coded by significance (red indicated *p* < 0.05).

Normal walking was positively associated with sensorimotor (SOM; *β*_adj_ = 0.31, *p* = 0.02) and dorsal attention (DAN; *β*_adj_ = 0.29, *p* = 0.03) network connectivity, such that the greater the within-network connectivity, the faster the participants walked at preferred pace. However, preferred walking speed was negatively associated with the visual network (VIS; *β*_adj_ = −0.31, *p* = 0.02). Walking while simultaneously performing the serial subtraction task (i.e., dual task) was positively associated with within-network SOM (*β*_adj_ = 0.43, *p* = 0.003), DAN (*β*_adj_ = 0.34, *p* = 0.03), and ventral attention (VAN; *β*_adj_ = 0.34, *p* = 0.03) networks. DTC was associated with the SOM (*β*_adj_ = −0.31, *p* = 0.03) network, such that the greater the within-network connectivity, the lower the cost (slowing) to gait speed during the DT. These associations are depicted in Figure 1.

#### Between-Network Associations

**Figure 2:**
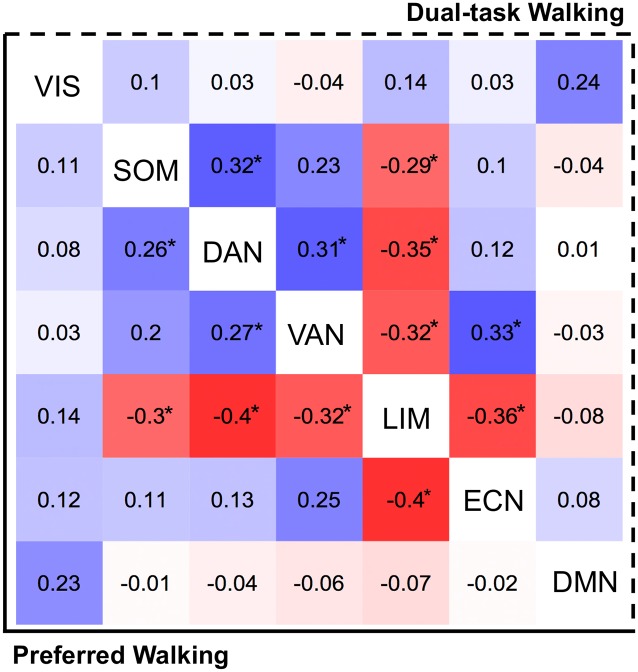
Heat map reflecting between-network fMRI connectivity associations (standardized betas) with preferred walking (left of diagonal) and walking while dual-tasking (right of diagonal). Tiles are color-coded by strength (positive = blue, negative = red). Associations exceeding *p* = 0.05 threshold (uncorrected) are presented with asterisk (*).

Figure 2 illustrates the associations of inter-network averages with preferred (lower triangle) and DT (upper triangle) walking speeds. These associations were found to be rather consistent across the two conditions (see Figure 2), which is not surprising since the walking speeds themselves were strongly correlated (*r* = 0.92, *p* < 0.0001). In both conditions, faster walking was associated with greater DAN connectivity with the SOM (*β*_adj_ > 0.26, *p* < 0.05) and the VAN (*β*_adj_ > 0.27, *p* < 0.04). Faster walking while performing the serial subtraction task was further associated with greater communication between the VAN and executive control network (ECN; *β*_adj_ = 0.33, *p* = 0.01). Interestingly, faster walking was also associated with lesser between-network limbic connectivity with motor and cognitive networks, including SOM, DAN, VAN, and ECN (*β*_adj_ < −0.29, *p* < 0.05). Lower DTC was solely associated with increased VAN-ECN connectivity (*β*_adj_ = −0.29, *p* = 0.04; not shown). No other significant between-network associations were found.

## Discussion

In the current study, we uncovered associations between neural network connectivity and walking speed in a sample of community-dwelling older adults from the MOBILIZE Boston Study. Specifically, we found that stronger cognitive and motor network connectivity was associated with faster walking in older adults, while greater communication with the limbic network was associated with slower walking. These findings were generally evident with and without a simultaneous task.

When we investigated individual differences in brain connectivity and preferred (i.e., quiet) walking speed, we found that faster walking speed was associated with increased connectivity within the motor and dorsal attention networks and decreased connectivity within the visual network. These findings align well with literature, as these networks include many of the cortical regions observed in task-based fMRI activation studies of gait speed, including the pre- and postcentral gyrus, inferior frontal gyrus, superior parietal lobes, and occipital areas (Hamacher et al., 2015). Further, stronger between-network connectivity (i.e., between motor and dorsal as well as ventral attention networks) was associated with faster walking. This provides evidence that these networks do not work in isolation, but rely upon one another in the engagement of motor, sensory and visual functions, as well as a balance of top-down (i.e., dorsal) and bottom-up (i.e., ventral) goal-directed attention (Corbetta and Shulman, 2002; Kincade et al., 2005). While we did not predict negative associations with the visual network, other studies suggest that this network has altered connectivity with older age (Goh, 2011; Geerligs et al., 2015), which could impact motor control and gait. In our DT paradigm, where individuals have to shift attention between serial subtraction and walking, we observed “further relationships” with the ventral attention network, suggesting a greater necessity for re-orienting of attention (Corbetta and Shulman, 2002). Reorienting of attention is potentially necessary for successful dual task performance, as supported by recent studies where VAN-DAN communication and VAN-DMN suppression were associated with better distractor suppression during visual search (Kelly et al., 2008; Poole et al., 2016). It should also be noted that the VAN spatially overlaps with the vestibular network identified in Yuan et al. (2015), which is also associated with walking speed during resting state fMRI.

The current study also provides evidence that the executive control network is associated with faster walking, especially during the DT condition. Previously, abnormal executive function, which is essential for attentional processes and the ability to plan, organize, and multi-task, had been linked with poorer dual tasking (Ohsugi et al., 2013), slower gait speed (Cohen et al., 2016), and falls (Mirelman et al., 2012) in older adults. While we have previously shown that walking speed is associated with ECN brain activation and structural connectivity (Jor’dan et al., 2017; Poole et al., 2018), we now suggest that how this network interacts with other neighboring networks may be essential to cognitive functioning in walking. To our knowledge, we are the first to explore all of these network relationships in cognitively healthy older adults.

On the other hand, slower walking was associated with greater widespread connectivity of the limbic network with motor and cognitive networks. The cortical areas contained in this network are associated with memory, arousal, and emotion (Agosta et al., 2012; Rolls, 2019). Prior imaging studies have found the limbic network to be hyper-connected in individuals with lesser motor expertise (Milton et al., 2004) and increased cognitive impairment (Badhwar et al., 2017). Thus, it is possible that increased communication with this network may lead to interference with motor and/or cognitive processes. It may also indicate active caution and/or fear while walking (Pannekoek et al., 2013).

### Clinical Implications and Future Directions

This study elucidates the underlying brain networks associated with preferred and DT walking performance, which are strong predictors of falls in older adults (Verghese et al., 2002; Quach et al., 2011). These network interactions could inform interventions that non-invasively target these brain networks (i.e., non-invasive brain stimulation) or their functions (i.e., motor-cognitive training) for individuals with mobility impairments. Further investigation of these networks may provide information on the progress of dysfunction, risk of falls, or the efficacy of rehabilitation.

However, these findings do not confirm the specific neural resources of utmost importance to mobility, especially in cases of “cognitive reserve” when alternate brain regions are recruited in the presence of structural degeneration (Stern, 2002; Venkatraman et al., 2010). Therefore, future studies should investigate the relative contributions of brain structure and function, as well as consider other clinical predictors of slow gait (Rosso et al., 2013). Furthermore, it is important to determine the reliability and generalizability of these findings in other older adult populations, in order to determine the best avenue for early intervention.

### Limitations

The current study has limitations. First, although this MOBILIZE Boston Study sample is a relatively small “representative” older adult population living with several comorbidities, previously collected Mini-Mental State Examination scores (administered within approximately 2 years of gait and MRI) indicate a range of cognitive health. To minimize the contributions of overt cognitive impairment to declines in walking speed, we only included those with a past MMSE of 25 or greater and no self-reported diagnosis of dementia. However, these results should be interpreted with caution since more recent cognitive measures were not collected.

Another limitation is that accuracy during the serial subtraction task was not explicitly emphasized in instructions, neither was it measured to allow calculation of a cognitive DTC. However, previous studies suggest a tendency for participants to prioritize focus on the cognitive task at the expense of the other (i.e., walking; Krampe et al., 2011). Nevertheless, without performance measures, it is unclear the degree to which serial subtraction task difficulty and attention prioritization contributed to the observed declines in gait speed without a baseline or report of math anxiety. As a result, we generally attribute slower walking during serial subtraction to multi-tasking, such that those with greater cognitive connections have greater ability to maintain walking speed while performing an additional task.

Finally, with regard to the rs-fMRI analysis, although many approaches exist [e.g., seed-based connectivity, independent component analysis (ICA), principal component analysis (PCA)], here we utilize a brain atlas from Schaefer et al. (2018) to parse the brain into 100 regions. This atlas was derived from local gradient and global similarity approaches and is co-registered with a widely used cortical atlas of seven functionally-connected networks (Yeo et al., 2011). The seven-network atlas, which was derived from an independent sample of 1,000 healthy individuals, is relatively coarse, but has been well characterized in health and disease. By parsing the brain according to the Schafer atlas, we are able to retain the ability to reduce the dimensionality of rs-fMRI data, and estimate functional connectivity within and across each brain network, while increasing the resolution of the aforementioned seven networks (though averaged into 28 within and between-network “pairs”). This approach also allowed us to emphasize network-level conclusions and compare our results to other studies of older adult mobility. However, future studies should consider using *surface-based* approaches and complementary structure analyses to investigate individual differences since volumetric approaches are not sensitive to the heterogeneity in brain anatomy, especially across older adults with varying degrees of brain atrophy (Long et al., 2012).

## Conclusion

This study reports associations between brain functional connectivity and walking outcomes in a sample of community-dwelling older adults. Rs-fMRI analyses revealed that walking at faster preferred speed is associated with stronger connectivity within and between sensorimotor and dorsal attention networks, networks associated with motor control and goal-directed attention. With an added serial subtraction dual task, we found that stronger connectivity between attention and executive networks is associated with faster walking speed. However, stronger connectivity between these networks and the limbic network is associated with slower walking during both tasks.

## Data Availability

The datasets generated for this study are available on request to the corresponding author.

## Ethics Statement

This study was carried out in accordance with the recommendations of the Hebrew SeniorLife and VA Boston Healthcare System institutional review boards. All subjects gave written informed consent in accordance with the Declaration of Helsinki.

## Author Contributions

VP: data processing, analysis, and interpretation; wrote the manuscript. O-YL: analysis, interpretation. TW and II: project management; acquisition of data. LL and ME: study concept and design, study supervision, critical revisions of the manuscript for important intellectual content.

## Conflict of Interest Statement

The authors declare that the research was conducted in the absence of any commercial or financial relationships that could be construed as a potential conflict of interest.
